# A Rare Case of Cutaneous Metastasis of Renal Cell Carcinoma to the Lateral Thigh

**DOI:** 10.7759/cureus.37457

**Published:** 2023-04-11

**Authors:** Michael McBride, Guy Charest, Niki Nourmohammadi, Donald Christensen, Anabel Goulding

**Affiliations:** 1 Dermatology, HonorHealth Dermatology Residency Program, Scottsdale, USA; 2 Medicine, Lake Erie College of Osteopathic Medicine, Greensburg, USA; 3 Pathology, Western and Pinnacle Pathology, Phoenix, USA; 4 Medicine, Western University of Health Sciences, Pomona, USA

**Keywords:** metastatic lesion, clear renal cell carcinoma, cutaneous metastasis, renal cell metastasis, renal cell carcinoma (rcc)

## Abstract

Renal cell carcinoma (RCC) is a common cancer type in the United States, and at the time of diagnosis, many patients already have metastatic disease. RCC typically metastasizes to the lungs, liver, and bones, with few cases manifesting cutaneous metastasis. Most incidences of RCC metastases reported in the literature have been on the face and scalp. We discuss a case of a 64-year-old male patient who presented with a history of RCC and a purpuric nodule on his lateral thigh. Histopathological examination revealed vacuolated cytoplasm with areas of cytoplasmic clearing; the cells stained positively for cytokeratin AE1/AE3, CAM5.2, and PAX8. Cutaneous metastatic RCC was subsequently diagnosed. Cutaneous manifestations of RCC, particularly to the thigh, remain a rare presentation of metastatic RCC.

## Introduction

Renal cell carcinoma (RCC) accounts for 90% of all renal cancers and approximately 2% of cancer deaths annually [1,2]. Incidence rates of RCC have increased over the past several decades [1]. At the time of diagnosis, approximately 30% of patients with RCC present with metastatic disease, most commonly occurring in the lungs, liver, or bones [2]. Cutaneous manifestations of RCC are rare, accounting for only 1-3.3% of RCC metastases [3]. Reports of cutaneous manifestations of metastatic RCC most frequently involve the scalp and face, followed by the chest and abdomen [4]. We present a unique case of cutaneous metastatic RCC to the lateral thigh. The thigh is an uncommonly reported location for RCC metastasis.

## Case presentation

**Figure 1:**
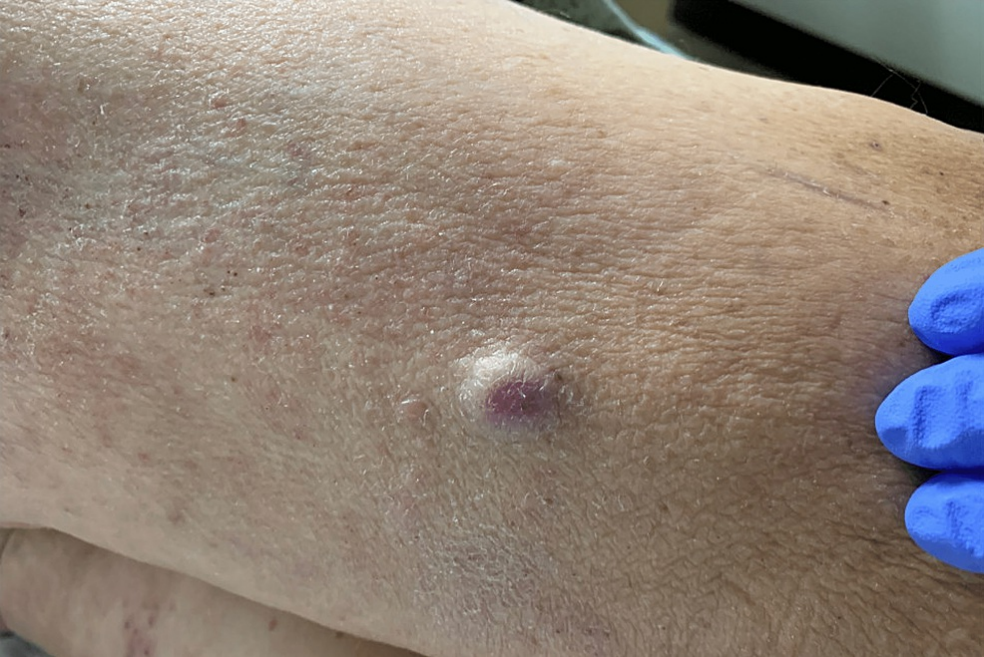
Right lateral thigh nodule

A 64-year-old male with a history of metastatic clear cell renal carcinoma to the right femur and spine presented to the emergency department for the evaluation of a rash on his lower extremities. The RCC had been diagnosed three years prior to the patient's hospital visit. His chemotherapy regimen consisted of Keytruda and axitinib. Dermatology was consulted to evaluate the patient for the diffuse rash on his lower extremities. Physical examination revealed diffuse palpable purpuric lesions on the lower extremities and a single blanching nodule on the right lateral thigh (Figure 1). Two 4-mm punch biopsies were obtained from these two distinct morphologies to help with the diagnosis.

**Figure 2:**
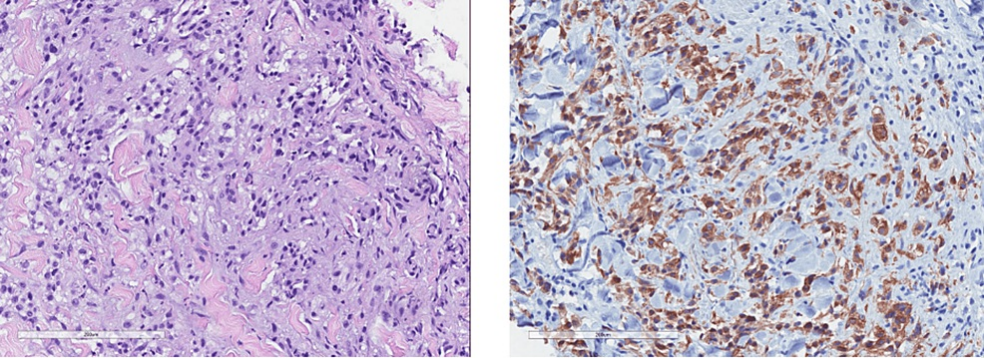
Histology from thigh nodule showing atypical cells with prominent nucleoli and moderate cytoplasm with cytoplasmic clearing; H&E and AE1-3 used respectively

Histology from the diffuse palpable purpuric lesions was consistent with leukocytoclastic vasculitis. These lesions were thought to be secondary to the patient's chemotherapeutic regimen, which had been changed a few months prior to rash development. Histology from the single blanching nodule revealed a dense dermal infiltrate of atypical cells with variably prominent nucleoli and moderate vacuolated cytoplasm with areas of cytoplasmic clearing (Figure 2). These cells stained positive for cytokeratin AE1/AE3, CAM5.2, and PAX8, and negative for CD163, RCC, CK7, CD10, and BerEP4. Given the clinical history and immunohistochemistry findings, this lesion was ultimately diagnosed as cutaneous metastatic RCC to the right lateral thigh. The patient opted for palliative care and passed away several weeks after hospitalization.

## Discussion

RCC commonly metastasizes to the lungs, liver, bones, lymph nodes, contralateral kidney, and adrenal glands [2]. In rare cases, RCC can exhibit metastatic disease to the skin, constituting 6% of all cutaneous metastases [5]. The most frequent locations of RCC cutaneous metastasis are the face and scalp [5].

Cutaneous metastatic RCC lesions clinically present as rapidly growing, painless nodular lesions [6]. The occurrence of these lesions typically indicates advanced disease and is associated with a poor prognosis with estimated life expectancy being less than one year [3,7]. As our case demonstrates, not all cutaneous metastatic lesions are predictable and other body sites can be involved. It is important to consider metastatic disease in the differentials in patients with RCC. The vascular appearance of these lesions leads to differential diagnoses that include hemangioma and pyogenic granulomas, and differentiation by histopathology is required for reaching a diagnosis [5].

Immunohistochemical markers can be helpful for the diagnosis of cutaneous metastatic RCC. PAX8 staining in a large series involving metastatic clear cell RCC patients reported high sensitivity (94%) and overall specificity (88%) [8]. Other studies have reported CD10 as a marker for metastatic clear cell RCC, with a sensitivity ranging from 83% to 100%; however, CD10 also stains positive in other non-renal tumors [9,10] Another immunohistochemical marker commonly used is renal cell carcinoma monoclonal antibody (RCCma or RCC). However, several recent studies have highlighted problems related to the specificity of the RCC marker since a variety of different tumors also stain positive for RCC [11,12]. Another marker commonly cited is cytokeratin 7 (CK7). Several studies have found CK7 negative or only occasionally focal positive in clear cell RCC, but not diffusely positive [13]. Given these studies showing high sensitivity and specificity of PAX8 and considering that CD10 and RCC are seen in a wide variety of non-renal neoplasms, some authors believe that PAX8 should replace both RCC and CD10 in routine diagnostic practice [14-18].

Given the significant morbidity and mortality associated with metastatic disease, it is critical for clinicians to be able to recognize these clinical lesions for a prompt diagnosis.

## Conclusions

The occurrence of RCC metastasis to the skin is rare. Unfortunately, at the time of presentation of these lesions, the cancer is typically in advanced stages. However, with appropriate surgical excision and treatment, disease-free follow-up is achievable. Therefore, RCC metastases should be included in the differential diagnosis of vascular-appearing nodules in patients with a history of renal cancer.
